# Incidence of endoleak type IA in patients undergoing chimney endovascular aortic repair (ChEVAR) vs. standard endovascular repair

**DOI:** 10.47487/apcyccv.v5i1.346

**Published:** 2024-03-19

**Authors:** Martin Rabellino, Juan Guido Chiabrando, Fernando Garagoli, María Marta Abraham Foscolo, María de los Milagros Fleitas, José Chas, Vanesa Di Caro, Ignacio Martin Bluro, Sergio Shinzato

**Affiliations:** 1 Departamento de Angiografía Digital, Hospital Italiano de Buenos Aires. Buenos Aires, Argentina. Departamento de Angiografía Digital Hospital Italiano de Buenos Aires Buenos Aires Argentina; 2 Departamento de Cardiología, Hospital Italiano de Buenos Aires. Buenos Aires, Argentina. Departamento de Cardiología Hospital Italiano de Buenos Aires Buenos Aires Argentina

**Keywords:** Techniques, Endovascular, Endovascular Aortic Repair, Endoleak, FEVAR, Procedimientos Endovasculares, Reparación Endovascular de Aneurismas, Endofuga, Reparación de Aneurisma Endovascular Fenestrado

## Abstract

**Objectives.:**

Almost half of endovascular aortic aneurysm repair (EVAR) procedures are performed in hostile anatomy, increasing the risk of procedure related complications such as type IA endoleaks, which may be prevented with the chimney technique in EVAR (ChEVAR). Our aim is to describe the differential characteristics between EVAR in favorable anatomy and ChEVAR in hostile necks.

**Materials and methods.:**

A cohort of patients with infrarenal abdominal aortic aneurysms (AAA) that were treated with EVAR or ChEVAR were included. The primary outcome was the incidence of type IA endoleak. Secondary outcomes were the rate of chimney occlusion, reintervention, migration, rupture, acute limb ischemia, sac growth, and aneurysm-related mortality during the follow-up period.

**Results:**

. With a median follow-up of 11.5 months, 79 patients were treated with EVAR and 21 with ChEVAR. The overall age was 76.49 ± 7.32 years old, and 82% were male. The ChEVAR cohort had a higher prevalence of tobacco use than the EVAR cohort (38.1% vs. 17.7%, p = 0.041), and a shorter neck (7.88 mm ± 5.73 vs 36.28 mm ± 13.73, p<0.001). There were no differences in type IA endoleak incidence between the groups (a single endoleak type IA in the EVAR group, p = 0.309). One patient experienced an asymptomatic chimney occlusion in the ChEVAR group, and another patient required a reintervention due to chimney occlusion. Sac regression and reinterventions were not different between groups. There were no migration, rupture, acute limb ischemia, or aneurysm-related mortality events.

**Conclusions:**

. In patients with abdominal aortic aneurysms, ChEVAR in hostile necks had similar event rates to EVAR in favorable necks.

## RESUMEN

**Objetivos.:**

Aproximadamente la mitad de las reparaciones endovasculares de aneurisma de aorta abdominal (AAA) son realizadas en anatomías hostiles, incrementando el riesgo de complicaciones como endoleaks tipo IA. La técnica con chimeneas (ChEVAR) es una alternativa para disminuir el riesgo de complicaciones en cuellos hostiles. Nuestro objetivo es comparar ambas técnicas (ChEVAR y reparación endovascular convencional [EVAR]) en nuestra medio.

**Materiales y métodos.:**

Se realizó un trabajo de cohorte retrospectivo en pacientes con AAA tratados con EVAR o ChEVAR. El punto final primario fue la incidencia de endoleak tipo IA. Los puntos finales secundarios fueron la incidencia de oclusión de chimeneas, reintervención, migración, ruptura del saco, isquemia aguda de miembros, crecimiento del saco o mortalidad asociada al aneurisma durante el seguimiento.

**Resultados.:**

Tras una mediana de seguimiento de 11,5 meses, 79 pacientes fueron tratados con EVAR y 21 con chEVAR. La edad promedio fue de 76,49 ± 7,32 años y 82% fueron de sexo masculino. Los pacientes con chEVAR tuvieron mayor prevalencia de consumo tabáquico que los pacientes con EVAR (38,1% vs. 17,7%, p=0,041) y un cuello más corto (7,88 mm ± 5,73 vs. 36,28 mm ± 13,73, p<0,001). No hubo diferencia de endoleak tipo IA entre los grupos. Dos pacientes presentaron la oclusión total de la chimenea, uno de los cuales requirió reintervención. No hubo diferencias en la regresión del tamaño del saco, así como tampoco hubo eventos de migración, ruptura, isquemia del miembro o mortalidad asociada al aneurisma.

**Conclusiones.:**

En pacientes con AAA, la técnica ChEVAR en cuellos hostiles tuvo eventos similares que EVAR en cuellos favorables.

## Introduction

Endovascular aneurysm repair (EVAR) became the preferred treatment for most patients with infrarenal abdominal aortic aneurysms (AAA) due to its less invasive nature, lower perioperative morbidity and mortality, and shorter hospital stay compared to open surgical repair ^1^^,^^2^. In order to provide a safe and effective treatment and prevent graft-related adverse events, anatomical characteristics are crucial. Neck length less than 10 mm, focal neck bulge greater than 3 mm, reverse taper greater than 2 mm within 1 cm below the renal arteries, neck thrombus or calcification more than 50% of the circumference, and angulation larger than 60 degrees within 3 cm below the renal arteries are all signs of a hostile neck ^3^. In fact, almost half of EVAR procedures are performed in hostile anatomy, mainly due to a short neck length, a greater angulation than recommended, and a larger neck diameter, ultimately providing conflicting data regarding long-term outcomes ^4^^,^^5^. 

This assumed risk, in order to avoid open surgery, may be decreased with a technique that offers a better abdominal aortic aneurysm seal, above the origin of the renal arteries and/or superior mesenteric artery, which can be protected with stent grafts. The endovascular alternative for this type of patient is fenestrated devices (FEVAR), whether custom-made or homemade, usually employed in patients with juxta renal, pararenal, or thoracoabdominal aneurysms and also in patients with hostile necks. In these patients (i.e., hostile neck), the chimney technique in endovascular aortic aneurysm repair (ChEVAR) involves the placement of a stent or stent-graft parallel to the main aortic stent-graft to extend the proximal or distal sealing zone while maintaining side branch patency ^6^^,^^7^. An advantage of ChEVAR compared to FEVAR is the availability in our country as the cost of the stents is lower compared to the custom or homemade devices. Moreover, although this technique was once thought to have limited follow-up, currently, the level of evidence is similar to that of fenestrated grafts, especially when these types of devices are not available ^8^.

As data are lacking on chEVAR and hostile and favorable neck EVAR respectively, we aim to describe the characteristics of hostile neck ChEVAR patients and favorable neck EVAR patients in our population.

## Materials and Methods

### Design

A retrospective cohort study at a university hospital in Argentina. Patients were included from January 2019 to June 2022 and followed up via a thorough chart review or, if no follow-up was reported by the third month, by telephone. The decision regarding the type and size of the prosthesis was subject to the operator’s preference. The decision to perform a standard EVAR or a ChEVAR was made according to the clinical expertise of the interventionist and the anatomical features of the patient. 

### Population

Male or female patients over 60 years old, with an asymptomatic AAA measuring at least 5.5 cm in any plane or with a growth larger than 10 mm in a year on a computed tomography (CT) scan, with favorable or hostile neck suitable for EVAR or ChEVAR, respectively. Both percutaneous and surgical accesses were included. Patients with ruptured, symptomatic but not ruptured, or inflammatory AAA were excluded from the analysis. We defined a hostile neck as a neck length less than 10 mm, focal neck bulge greater than 3 mm, reverse taper greater than 2 mm within 1 cm below the renal arteries, neck thrombus or calcification more than 50% of the circumference, and angulation larger than 60 degrees within 3 cm below the renal arteries.

### Procedures

CT evaluation: Factors that were accounted for in the pre-procedural CT included: the axial length from the aneurysm neck (distance between the lowermost renal artery and the start of the aneurysmal dilation), the shape and angulation of the neck, the diameter of the iliac arteries (for access through the groin), and the potential length and condition of the distal arteries used for two fixations of the device. In addition, we accounted for thrombosis, calcification, or tortuosity present at the intended sites of fixation.

A post-procedure control CT was performed at one month in patients undergoing endovascular repair and annually thereafter.

EVAR technique: A bilateral anterior echo-guided common femoral artery puncture was performed. We performed pre-closure with two percutaneous closure devices (Proglide) in each femoral access before inserting the main branch and the side branch of the endoprosthesis, which were implanted according to the instructions for use. 

ChEVAR technique: the technique is similar to the EVAR technique with the addition of the implant of a stent graft parallel to the main aortic stent graft to extend the proximal or distal sealing zone while maintaining side branch patency. If less equal than two chimneys were planned, a percutaneous approach was performed, with a 6F access in both humeral arteries. If three chimneys were needed, an open surgical subclavian access was performed.

### Outcomes

The primary outcome was the incidence of type IA endoleak after ChEVAR or EVAR. Secondary outcomes were the rate of chimney occlusion, reintervention, migration, endoleak (type III and IV endoleaks were excluded), rupture, acute limb ischemia, sac growth, and aneurysm-related mortality during the follow-up period ^5^. Furthermore, technical success (defined as a procedure completed as intended, with no secondary procedures within 30 days), abdominal aortic aneurysm sac diameter regression and all-cause mortality were analyzed as well.

### Definition of variables

Reintervention: requirement of additional procedures in order to complete the exclusion of the AAA sac. Migration: modification of the ChEVAR or EVAR anchoring site during the procedure or when evaluating a post-procedure CT. Endoleak Type IA: perigraft leak, perigraft channel, or graft-related endoleak at the proximal end of the graft. Endoleak Type IB: perigraft leak, perigraft channel, or graft-related endoleak at the distal end of the graft. Endoleak Type II: retrograde endoleak, collateral flow, retroleak, or non-grade related endoleak. Leak from the patient’s lumbar, inferior mesenteric, or intercostal arteries. Endoleak Type III: fabric tear, modular disconnection or poor seal, stent frame fracture or separation, attachment system fracture in addition to endoleaks. Endoleak Type IV: secondary to graft porosity and is typically seen in the immediate postoperative angiogram following an endovascular aneurysm repair ^9^. 

### Statistical analysis

Categorical variables are expressed as counts and percentages and analyzed with the Chi-squ 

are test or Fisher test, as appropriate. Continuous variables are expressed as mean ± standard deviation, median, and interquartile range (IQR), according to the distribution, and compared with the T-test or Mann-Whitney test, as appropriate. Moreover, we performed a multivariable logistic regression analysis in order to account for clinically relevant variables (such as sex, angulation, serum creatinine, neck diameter, and body mass index [BMI]) that may bias the relationship between the comparison of ChEVAR and EVAR regarding the primary outcome, providing the odds ratio (OR) with 95% confidence intervals (CI). 

Time-to-mortality estimates were made using a multivariable Cox regression, and hazard ratios (HR) were reported. The reference category for HRs was patients treated with EVAR. All statistical tests were two-tailed, and P values <0.05 were considered to indicate statistical significance. All analyses were performed with R 4.0.3 (R Foundation for Statistical Analysis, Vienna, Austria).

### Ethical considerations

Due to the retrospective nature of this study, an exemption from informed consent was deemed necessary. The manuscript received approval from our institutional review board. All of our work follows the Declaration of Helsinki and good clinical practice guidelines. 

## Results

With a median follow-up of 11.5 months (IQR: 4.1 - 24.8), a total of 100 patients met the inclusion criteria: 79 patients were treated with EVAR and 21 with ChEVAR. The overall age was 76.49 ± 7.32 years old, and 82% were male. The ChEVAR cohort had a higher frequency of tobacco use than the EVAR cohort (38.1% vs. 17.7%, p = 0.041). A good balance existed between the groups for other cardiovascular comorbidities. The majority of the tomographic characteristics (such as thrombus or calcium >50%, sacculation >3 mm, proximal and distal neck diameters, and angulation) were comparable between cohorts with the exception of the neck length, which was shorter in the ChEVAR cohort patients compared to the EVAR cohort patients (7.88 ± 5.73 mm vs. 36.28 ± 13.73 mm, p<0.001) (Table 1).

**Table 1:** Baseline characteristics

Baseline Characteristics	Overall n = 100	EVAR n = 79	ChEVAR n = 21	p-value
Age (years)*	76.49 ± 7.32	76.58 ± 7.28	76.15 ± 7.65	0.811
Male (%)	82 (82.0)	64 (81.0)	18 (85.7)	0.858
Weight (kg)*	82.63 ± 14.91	81.67 ± 13.18	86.24 ± 20.15	0.214
Height (mts)*	1.71 ± 0.09	1.71 ± 0.08	1.74 ± 0.09	0.131
BMI*	28.10 ± 4.33	28.05 ± 3.98	28.32 ± 5.57	0.798
Arterial hypertension (%)	76 (76.0)	62 (78.5)	14 (66.7)	0.401
Diabetes Mellitus (%)	15 (15.0)	12 (15.2)	3 (14.3)	1
Chronic Kidney Disease (%)	15 (15.0)	9 (11.4)	6 (28.6)	0.106
Active tobacco consumption (%)	22 (22.0)	14 (17.7)	8 (38.1)	0.041
Peripheral vascular disease (%)	12 (12.0)	8 (10.1)	4 (19.0)	0.459
Coronary artery disease (%)	26 (26.0)	18 (22.8)	8 (38.1)	0.254
Stroke (%)	11 (11.0)	8 (10.1)	3 (14.3)	0.881
Atrial fibrillation (%)	12 (12.0)	9 (11.4)	3 (14.3)	1
Previous LV Ejection Fraction **	55% [55 - 61]	55 % [55 - 61]	55% [55 - 60]	0.562
Neck Tomographic Characteristics	n=91	n=71	n=20	
Thrombus or Calcium >50% (%)	33 (36.3)	28 (39.4)	5 (25.0)	0.356
Saculation > 3mm (%)	5 (5.5)	3 (4.2)	2 (10.0)	0.656
Proximal Neck Diameter (mm)*	23.37 ± 4.07 (n=90)	23.23 ± 3.13 (n=70)	23.88 ± 6.45	0.526
Distal Neck Diameter (mm)*	25.66 ± 4.43	25.46 ± 4.10	26.34 ± 5.51	0.434
Maximal Neck Diameter (mm)*	26.66 ± 4.76	26.36 ± 4.44	27.73 ± 5.76	0.257
Neck Length (mm)*	30.04 ± 17.13	36.28 ± 13.73	7.88 ± 5.73	<0.001
Angulation (degrees)*	57.77 ± 58.78 (n=89)	59.86 ± 61.54 (n=69)	50.53 ± 48.73	0.535

* mean ± standard deviation

** median [IQR]

BMI: body mass index. LV: left ventricular

The Endurant Medtronic prosthesis was the most frequently used (81% vs. 64.5% in the ChEVAR and EVAR groups, respectively) with no differences between the groups (p=0.15). A total of 43 chimneys were implanted using only balloon expandable covered stents (BECS), with the most common technique being the implantation in both renal arteries (71.4%), followed by both renal arteries and the superior mesenteric artery (3 patients, 14.3%), only 1 renal artery (2 patients, 9.5%) and finally 1 renal artery with the superior mesenteric artery (1 patient, 4.7%). 

We found no differences between the primary endpoint of endoleak type IA between the groups (1 endoleak type IA in the EVAR group). Type II endoleaks were the most frequent type overall (incidence of 21%), with no differences between the groups (14.3% in ChEVAR vs. 22.8% in EVAR, p = 0.583) (Table 2). 

**Table 2:** Clinical endpoints.

Endpoints	Overall n = 100	EVAR n = 79	ChEVAR n = 21	p-value
All Cause Death (%) *	9 (9.0)	6 (7.6)	3 (14.3)	0.601
Endoleak (%)	23 (23)	19 (26.8)	4 (19)	0.561
Type IA	1 (1)	1 (1.3)	0 (0)	0.301
Type II	21 (21)	18 (22.8)	3 (14.3)	0.583
Reintervention (%)	1 (1.0)	0 (0.0)	1 (4.8)†	0.474
Chimney Occlusion (%)	2 (2)	-	2/43 (2.3) ‡	NA
Aortic Sac diameter difference **	-4.17 ± 10.32	-4.52 ± 10.90	-2.91 ± 8.04	0.562
Length of stay **	2.13 ± 1.86	1.97 ± 1.64	2.71 ± 2.49	0.106
Follow up (months) **	11.5 ± 13.65	17.58 ± 14.01	9.11 ± 9.85	0.011

† Reintervention due to chimney occlusion after antiplatelet suspension due to bleeding.

‡ The occlusion occurred at a left renal artery (Begraft Stent 6.0 x 38 mm)

*All deaths were not related to the procedure

** Mean ± standard deviation

NA not aplicable

One patient experienced an asymptomatic chimney occlusion detected in a routine CT, and only one patient required a reintervention in the ChEVAR group due to chimney occlusion after anti-platelet therapy suspension due to intestinal bleeding. The primary and secondary chimney permeabilities were 93% and 97.7%, respectively. We found no migration, rupture, or acute limb ischemia in any of the groups. Sac regression diameters were not different (-1.75 mm, [-3.88; -0.20] vs. -2.4 mm [-5.7; 0], p=0.779) in both ChEVAR and EVAR groups, as was the length of stay (1 day [1-2] vs. two days [1-3], p = 0.136). All-cause mortality during the follow-up was not different between the ChEVAR and EVAR groups (14.3% vs. 7.6%, respectively, p = 0.601), with no aneurysm-related mortality present. Of note, we report 100% success in both groups. 

After adjusting for angulation and neck diameter, we found no difference in the multivariable regression analysis regarding the primary outcome between the groups (OR 14; 95%CI: 0.5 - 53, p = 0.190) (Table 3). Finally, patients treated with ChEVAR did not have an increased risk of mortality compared to EVAR patients (HR 3.2; 95%CI:0.8 - 13, p = 0.09).

**Table 3:** Multivariable logistic regression analysis.

Variable	OR	95%CI	p-value
ChEVAR	4.25	0.6 - 55	0.320
Angulation	1.01	0.9 - 1.02	0.653
Maximum neck diameter	0.9	0.6 - 1.3	0.677

OR: odds ratio. CI: confidence interval

## Discussion

In our study, we found that patients treated with ChEVAR in hostile necks were not associated with an increased risk of type IA endoleak compared to EVAR in favorable anatomy in patients with aortic abdominal aneurysm. We also found that chimney occlusions were an extremely rare event, with only one patient having an asymptomatic chimney occlusion in a renal artery (incidence of 2.3%), and another patient that required a reintervention due to acute kidney injury due to a chimney occlusion. We found no other differences in survival, re-interventions, length of stay, or aortic sac regression between ChEVAR and EVAR patients. As far as we know, we report the first study comparing ChEVAR in hostile necks with EVAR in favorable necks.

For patients with AAA, EVAR has many remarkable benefits compared to traditional surgery, such as reduced trauma, shorter hospital stays, faster recovery, and lower perioperative morbidity and mortality rates, being particularly advantageous for patients who are considered high-risk for open surgery ^10^^,^^11^.

With improved technical skills and device-related refinements, EVAR has progressively expanded to more complex anatomies, frequently outside the manufacturer’s instructions for use in 41.9 to 69% of patients ^4^. Our results are in line with these, as 50% of our patients had at least 1 of the characteristics describing a hostile neck, with 29% having a large angulation, 39.4% having >50% thrombus or calcification circumference, and 4.2% having a sacculation >3 mm. All patients with a neck length of <10 mm were treated with ChEVAR. Especially in these patients with a very short neck, ChEVAR seems a very attractive strategy, as previous studies have associated type IA endoleaks with an increased risk of late rupture, open conversion, and death in patients treated with EVAR ^12^^,^^13^.

Although ChEVAR is a more complex procedure than EVAR, it leads to the preservation of visceral branches that arise from or are close to the aneurysm as well as a reduction of type IA endoleaks, broadening the spectrum of treatment for patients with abdominal aortic aneurysms. Indeed, since the first publications in 2003 and 2007 and the multicenter registry PERICLES, the use of snorkels and chimneys has evolved from a bailout strategy to a planned strategy to overcome the incidence of Type IA endoleaks in EVAR with hostile necks, providing a primary patency of 94%, and a secondary patency of 95.3%. These results were confirmed in an extended follow-up of the PERICLES registry, demonstrating a primary patency of the stent-grafts of 90.5% at 5 years ^14^^,^^15^. 

Furthermore, our chimney patency is in line with more recent studies such as the PROTAGORAS and PROTAGORAS 2.0 studies in which only balloon expandable stents were used ^16^^,^^17^. These studies allowed us to determine which type of device and stent-grafts were best suited for performing this technique, what level of oversizing to use, and the objectives that one should aim for, such as the total length of the new neck, in order to achieve proper sealing and reduce the gutters responsible for endoleaks. Indeed, we had no type IA endoleak, meaning that we improved the technique in order to seal the short neck, compared to the 2.9% type IA endoleak incidence present in the PERICLES registry ^14^. Although one of the caveats of the ChEVAR technique is the perceived increased risk of early gutter-related type IA endoleaks, they appear to resolve spontaneously in the majority of cases during early to midterm follow-up with no clinically relevant adverse outcomes ^18^.

One key concern is that previous studies associated the need for more than two stent grafts are related to a decrease in the primary patency ^8^. Most of our patients required chimneys to both renal arteries (71.4%), and only 19.2% required an additional chimney to the superior mesenteric artery. We report a particularly good primary patency of the stent grafts, perhaps related to the fact that we used only balloon expandable (BE) covered stents. The PROTAGORAS study determined that the best combination is nitinol endoprosthesis and BE-covered stents ^16^^,^^17^. Only one patient had an asymptomatic chimney occlusion, detected in a 3-month control CT angiography, and another patient required a reintervention in the ChEVAR group due to chimney occlusion after anti-platelet therapy suspension due to intestinal bleeding. In contrast, the PERICLES registry used BE-covered stents in 49.2% of patients, 39.6% were self-expanding covered stents, and the remaining were bare metal stents. 

As a tertiary center in Argentina, we have increased our experience in hostile aneurysmatic anatomy, treating patients with short necks systematically with ChEVAR. We performed a systematic fast track in both EVAR and ChEVAR groups that included bilateral percutaneous access, post-procedural extubating, and 24-hour intensive care monitoring with a next-day hospital discharge. This protocol could be performed on almost all patients in both groups, without differences between groups.

A key point that has not yet been discussed is the classification of aneurysms, particularly regarding their necks. In the Protagoras study, the classification of aneurysms was based on the type of open surgery the patient should receive, rather than on the characteristics of the aneurysm neck in cases where the neck was present ^16^. In our opinion, this is a crucial point that Protagoras 2.0 later confirms that the only factor preventing endoleak is the presence of an infrarenal neck ^17^. In our series, the average length of necks in the ChEVAR group was 7.88 mm, and no patient presented an immediate or late type IA endoleak, resulting in a 0% rate of type IA endoleaks. This is an important point to define because we cannot compare a juxta renal aneurysm with no neck to a patient with at least 5 mm of the infrarenal neck, as those 5 mm are sufficient to create a seal, and perhaps in that patient, it is not necessary to achieve a total length of the new neck >20 mm, as recommended by this study.

Nevertheless, to date, as there are no large RCTs supporting ChEVAR over surgical treatment in patients with severely short or absent necks, an alternative to ChEVAR remains fenestrated EVAR (FEVAR), with some observational studies providing comparable outcomes to ChEVAR with regard to technical success, target branch vessel patency, early mortality, type IA endoleak, postoperative renal dysfunction, or the need for secondary intervention. However, we believe that FEVAR is a more complex, technically demanding, and expensive procedure than ChEVAR due to the requirement of specialized equipment and techniques, often requiring longer hospital stays ^19^. Although we did not make a comparison between ChEVAR and FEVAR, we believe that ChEVAR seems like a more promising approach when treating short necks in patients with AAA. Indeed, the aim of our study is to compare ChEVAR in hostile necks with EVAR in favorable necks. Previous studies have shown an increase in type IA endoleaks with EVAR in hostile necks of almost 20% ^21^. Furthermore, the reason why a high percentage of renal (two vessels) chimneys were used is the fact that in ChEVAR the advantage of sealing in the hostile neck is that, as you deploy above the renal arteries, the entire neck is utilized, sealing the AAA sac. The use of chimney grafts in most mesenteric arteries was mainly because they arose practically at the level of the renal arteries, otherwise, there is no need to go beyond the renal arteries because the sealing is done below them in the hostile neck. Also, we consider that the number of chimney grafts does not affect the incidence or risk of type IA endoleaks because the sealing occurs in the neck of the aneurysm, which, although hostile, is present and sufficient for sealing. This concept is different in patients with juxtarenal aneurysms, where a new AAA neck for sealing is needed.

Certain limitations arise from our study. First, as we provide observational data, some unknown confounding variables cannot be fully accounted for. Second, we report only the last 100 AAA patients treated percutaneously, a sample size small enough to provide a precise estimate of the effect size; thereby, our results may not be generalizable to a larger population. Third, as our study is retrospective and data was previously collected, it may be subject to information bias. In retrospective studies, the data collection is based on existing records or databases, which may introduce selection bias. It is crucial to acknowledge that the study sample may not be representative of the entire population or may include a specific subgroup, potentially limiting the generalizability of the findings. Further studies are needed in order to provide generalizability to other populations. Finally, as we had a small sample size, sensitivity analysis such as EVAR in hostile necks cannot be performed. Larger studies will be necessary in order to elucidate this.

In conclusion, in patients with abdominal aortic aneurysms and hostile necks, the percutaneous approach with ChEVAR in hostile necks had similar event rates to EVAR in favorable necks. There were no differences between the two groups in terms of type IA endoleaks, we found no migration, rupture, acute limb ischemia, or aneurysm-related mortality during the follow-up period. Additionally, endoleak chimney occlusions were an extremely rare event, with only one patient having an asymptomatic chimney occlusion and another patient requiring a reintervention due to a chimney occlusion. Future studies are needed in order to confirm these results.

## References

[1] Greenhalgh RM, Brown LC, Kwong GPS, Powell JT, Thompson SG, EVAR trial participants (2004). Comparison of endovascular aneurysm repair with open repair in patients with abdominal aortic aneurysm (EVAR trial 1), 30-day operative mortality results randomised controlled trial. Lancet Lond Engl.

[2] Greenhalgh RM, Brown LC, Powell JT, Thompson SG, Epstein D, United Kingdom EVAR Trial Investigators (2010). Endovascular versus open repair of abdominal aortic aneurysm. N Engl J Med.

[3] Dillavou ED, Muluk SC, Rhee RY, Tzeng E, Woody JD, Gupta N (2003). Does hostile neck anatomy preclude successful endovascular aortic aneurysm repair. J Vasc Surg.

[4] Walker J, Tucker LY, Goodney P, Candell L, Hua H, Okuhn S (2015). Adherence to endovascular aortic aneurysm repair device instructions for use guidelines has no impact on outcomes. J Vasc Surg.

[5] Herman CR, Charbonneau P, Hongku K, Dubois L, Hossain S, Lee K (2018). Any nonadherence to instructions for use predicts graft-related adverse events in patients undergoing elective endovascular aneurysm repair. J Vasc Surg.

[6] Patel RP, Katsargyris A, Verhoeven ELG, Adam DJ, Hardman JA (2013). Endovascular aortic aneurysm repair with chimney and snorkel grafts indications, techniques and results. Cardiovasc Intervent Radiol.

[7] Bryce Y, Kim W, Katzen B, Benenati J, Samuels S (2018). Outcomes over Time in Patients with Hostile Neck Anatomy Undergoing Endovascular Repair of Abdominal Aortic Aneurysm. J Vasc Interv Radiol JVIR.

[8] Wanhainen A, Verzini F, Van Herzeele I, Allaire E, Bown M, Cohnert T (2019). Editor's Choice - European Society for Vascular Surgery (ESVS) 2019 Clinical Practice Guidelines on the Management of Abdominal Aorto-iliac Artery Aneurysms. Eur J Vasc Endovasc Surg Off J Eur Soc Vasc Surg.

[9] Chaikof EL, Dalman RL, Eskandari MK, Jackson BM, Lee WA, Mansour MA (2018). The Society for Vascular Surgery practice guidelines on the care of patients with an abdominal aortic aneurysm. J Vasc Surg.

[10] Patel R, Sweeting MJ, Powell JT, Greenhalgh RM, EVAR trial investigators (2016). Endovascular versus open repair of abdominal aortic aneurysm in 15-years' follow-up of the UK endovascular aneurysm repair trial 1 (EVAR trial 1) a randomised controlled trial. Lancet Lond Engl.

[11] Yokoyama Y, Kuno T, Takagi H (2020). Meta-analysis of phase-specific survival after elective endovascular versus surgical repair of abdominal aortic aneurysm from randomized controlled trials and propensity score-matched studies. J Vasc Surg.

[12] Tan TW, Eslami M, Rybin D, Doros G, Zhang WW, Farber A (2016). Outcomes of patients with type I endoleak at completion of endovascular abdominal aneurysm repair. J Vasc Surg.

[13] Fransen G.A.J, Vallabhaneni SR, van Marrewijk CJ, Laheij RJF, Harris PL, Buth J (2003). Rupture of Infra-renal Aortic Aneurysm after Endovascular Repair: A Series from EUROSTAR Registry. Eur J Vasc Endovasc Surg Off J Eur Soc Vasc Surg.

[14] Donas KP, Lee JT, Lachat M, Torsello G, Veith FJ, PERICLES investigators (2015). Collected world experience about the performance of the snorkel/chimney endovascular technique in the treatment of complex aortic pathologies the PERICLES registry. Ann Surg.

[15] Taneva GT, Lee JT, Tran K, Dalman R, Torsello G, Fazzini S (2021). Long-term chimney/snorkel endovascular aortic aneurysm repair experience for complex abdominal aortic pathologies within the PERICLES registry. J Vasc Surg.

[16] Donas KP, Torsello GB, Piccoli G, Pitoulias GA, Torsello GF, Bisdas T (2016). The PROTAGORAS study to evaluate the performance of the Endurant stent graft for patients with pararenal pathologic processes treated by the chimney/snorkel endovascular technique. J Vasc Surg.

[17] Fazzini S, Martinelli O, Torsello G, Austermann M, Pipitone MD, Torsello GF (2021). The PROTAGORAS 2 0 Study to Identify Sizing and Planning Predictors for Optimal Outcomes in Abdominal Chimney Endovascular Procedures. Eur J Vasc Endovasc Surg Off J Eur Soc Vasc Surg.

[18] Ullery BW, Tran K, Itoga NK, Dalman RL, Lee JT (2017). Natural history of gutter-related type Ia endoleaks after snorkel/chimney endovascular aneurysm repair. J Vasc Surg.

[19] Katsargyris A, Oikonomou K, Klonaris C, Topel I, Verhoeven ELG (2013). Comparison of outcomes with open, fenestrated, and chimney graft repair of juxtarenal aneurysms are we ready for a paradigm shift?. J Endovasc Ther Off J Int Soc Endovasc Spec.

[20] Donas KP, Torsello G, Bisdas T, Osada N, Schonefeld E, Pitoulias GA (2012). Early outcomes for fenestrated and chimney endografts in the treatment of pararenal aortic pathologies are not significantly different a systematic review with pooled data analysis. J Endovasc Ther Off J Int Soc Endovasc Spec.

[21] Mathlouthi A, Locham S, Dakour-Aridi H, Black JH, Malas MB (2020). Impact of suprarenal neck angulation on endovascular aneurysm repair outcomes. J Vasc Surg.

